# Remineralization and repair of enamel surface by biomimetic Zn-carbonate hydroxyapatite containing toothpaste: a comparative *in vivo* study

**DOI:** 10.3389/fphys.2014.00333

**Published:** 2014-09-05

**Authors:** Marco Lelli, Angelo Putignano, Marco Marchetti, Ismaela Foltran, Francesco Mangani, Maurizio Procaccini, Norberto Roveri, Giovanna Orsini

**Affiliations:** ^1^Department of Chemistry “G. Ciamician,” Alma Mater Studiorum, University of BolognaBologna, Italy; ^2^Department of Clinical Sciences and Stomatology, Polytechnic University of MarcheAncona, Italy; ^3^Department of Translational Medicine, Tor Vergata University of RomeRome, Italy

**Keywords:** enamel, toothpaste, Zn-carbonate hydroxyapatite nanocrystals, fluoride, repair, remineralization

## Abstract

Consumption of acidic foods and drinks and other factors that cause enamel wear are responsible for the daily enamel loss and degradation. Use of some toothpastes that have been showed to possess different properties of remineralisation and/or repair of the enamel surface may help to protect tooth enamel. The aim of this study was to evaluate whether the use of toothpaste containing Zn-carbonate hydroxyapatite (CHA) nanostructured microcrystals may exert remineralization/repair effects of the enamel surface. Two groups of patients, aged between 18 and 75 years, used a Zn-CHA nanocrystals-based toothpaste (experimental group) and a potassium nitrate/sodium fluoride toothpaste (active control group) for 8 weeks. At the end of this period, extractions were performed in five subjects per study group. Negative controls consisted of two subjects treated with non-specified fluoride toothpaste. Teeth were processed for morphological and chemical-physic superficial characterizations by means of Scanning Electronic Microscopy with Elementary analysis, X-Ray Diffraction analysis and Infrared analysis. In this study, the use of a Zn-CHA nanocrystals toothpaste led to a remineralization/repair of the enamel surface, by deposition of a hydroxyapatite-rich coating. On the other hand, the use of both a nitrate potassium/sodium fluoride and non-specified fluoride toothpastes did not appreciably change the enamel surface. In conclusion, this study demonstrates that the toothpaste containing Zn-CHA nanostructured microcrystals, differently from nitrate potassium/sodium fluoride and non-specified fluoride toothpastes, may promote enamel superficial repair by means of the formation of a protective biomimetic CHA coating.

## Introduction

Enamel loss as a part of tooth wear has become a common problem in everyday dental practice, which can be caused by erosion (Hooper et al., 2003), attrition and abrasion, being often associated with dentine hypersensitivity (DH) in different teeth (Absi et al., 1987; Addy, 1990; Addy et al., 2007; Addy and West, 2013). The predicted percentage of adults presenting severe tooth wear has increases with age from 3% at the age of 20 years to 17% at the age of 70 years (Van't Spijker et al., 2009).

Erosion is currently believed to be the major factor involved in tooth wear, and has been defined as the dissolution of teeth natural hydroxyapatite by either extrinsic or intrinsic acids, which are not originated from bacteria (Scheutzel, 1996; Zero and Lussi, 2000). Attrition describes the wear of teeth at sites of direct contact between teeth, which can be associated with occlusal function and may be exaggerated by parafunctional habits such as bruxism (Smith and Knight, 1984). Abrasion describes the wear of teeth caused by objects, including toothbrush, abrasive toothpastes as well as a variety of habits like pipe smoking (Dababneh et al., 1999). The combined action of all the above mentioned mechanisms may occur frequently, resulting in the dentine exposure, which might lead to the development of DH by means of the most widely accepted hydrodynamic theory proposed by Brannström (Brannström, 1963; Hefferren, 1976; Groeneveld et al., 1990; Drisko, 2007).

Among the numerous treatment regimens that have been recommended over these years, particular attention has been focused on toothpastes containing potassium salts (Markowitz et al., 1991; Nagata et al., 1994; Peacock and Orchardson, 1995; Orchardson and Gillam, 2000; Poulsen et al., 2006; Bellamy et al., 2009) and, more recently, on a new toothpaste formulation containing zinc (Zn)-substituted carbonate-hydroxyapatite (CHA) nanostructured microcrystals (from now on called Zn-CHA toothpaste), which has been shown to produce *in vitro* remineralization of the altered enamel surfaces and to be effective in closing dentinal tubules (Kuroiwa et al., 1994; Rimondini et al., 2007; Lee et al., 2008; Roveri et al., 2008, 2009a). Indeed, a clinical randomized trial has shown the efficacy of CHA-based toothpastes in reducing DH, after 4 and 8 weeks. In particular, this study demonstrated that Zn-CHA toothpaste compared with potassium nitrate/fluoride toothpaste (KNO_3_/NaF, active control) showed a significant improvement in airblast test scores (mean percentage of reduction 46.0 vs. 29.4% in controls) and subjective test scores (47.5 vs. 28.1%, respectively), with both differences being significant already after 4 weeks (Orsini et al., 2010).

However, this latter trial presented two major limitations consisting in: (a) the true efficacy of both toothpastes could not be assessed, since no morphological analyses were carried out on the dental surfaces; (b) no negative control such as a fluoride toothpaste (Holland et al., 1997) was used. Therefore, the aim of the present study was to investigate (1) whether the *in vitro* action of Zn-CHA toothpaste, based on the gradual remineralizing/repairing action of the dental surface by means of the deposition of a biomimetic CHA coating (Rimondini et al., 2007; Roveri et al., 2009a) could be confirmed also *in vivo*, and (2) whether the Zn-CHA toothpaste and the KNO_3_/NaF toothpaste may lead to stable morphological changes of the dental surface. These aims will be assessed by comparing the enamel surface of teeth treated *in vivo* for 8 weeks with: (I) Zn-CHA toothpaste (experimental group); (II) KNO_3_/NaF toothpaste (active control group); (III) fluoride toothpaste (negative control group).

## Materials and methods

### Study design and population

This study was carried out as the conclusive part of a previously published randomized trial conducted at the Department of Clinical Sciences and Stomatology of the Polytechnic University of Marche, Ancona, Italy (registered in the Australian New Zealand Clinical Trials Registry with the number: 00362190). According to Silverman et al. (1996) inclusion criteria were: hypersensitive area on facial surfaces of the teeth (incisors, cuspids, bicuspids and first molars with exposed cervical dentine), with at least two teeth scoring one or more at the air blast sensitivity test; good periodontal health (no probing depth >4 mm) with no other conditions which might explain their apparent DH; good physical health; age between 18 and 75 years; provision of written informed consent. Exclusion criteria were: chipped teeth, defective restorations, fractured undisplaced cuspids, deep dental caries or large restorations showing pulpal response, deep periodontal pockets, orthodontic appliances, dentures, or bridgework that would interfere with the evaluation of hypersensitivity; periodontal surgery within the previous 6 months; ongoing treatment with antibiotics and/or anti-inflammatory drugs; ongoing treatment for tooth hypersensitivity; pregnancy or lactation (Singal et al., 2005); acute myocardial infarction within the past 6 months, use of a pace-maker, uncontrolled metabolic diseases, major psychiatric disorder, heavy smoking and alcohol or drug abuse.

Eligible subjects were randomized to receive either the new toothpaste formulation (experimental group), containing biomimetic nanocrystals of Zn-substituted CHA, assembled in microparticles (Zn-CHA toothpaste, BioRepair® Plus, Coswell S.p.A., Funo, Bologna, Italy) or a commercially available desensitizing toothpaste (active control group), containing 5% KNO_3_/NaF with 1450 ppm fluoride (KNO_3_/NaF toothpaste, Sensodyne ProNamel™, GlaxoSmithKline Consumer Healthcare, Brentford, U.K.). The final protocol was approved by the Ethical Committee of the Polytechnic University of Marche, Ancona, Italy.

The clinical examination of the subjects was performed at baseline, after 4 weeks and after 8 weeks (end of the follow-up). During the visits, a minimum of two and up to four hypersensitive teeth was assessed using the most common and validated stimuli tests: tactile test, airblast test, cold water test and subjective test (Tarbet et al., 1979; Holland et al., 1997; Singal et al., 2005; Orsini et al., 2010).

Among the patients that completed the study, five subjects in the experimental group and five subjects in the active control group were selected for the present study because they needed extractions of sound teeth for orthodontic or prosthetic reasons. The extractions were performed after the end of the follow-up (8 weeks). At this time, two further subjects that needed extractions and brushed their teeth using a non-specified fluoride toothpaste were also included (fluoride toothpaste, negative control group).

### Analysis of Zn-substituted carbonate-hydroxyapatite (CHA) nanocrystals and CHA microclusters contained in the experimental toothpaste

Plate-acicular shaped CHA nanocrystals, about 70-100 nm in size, were synthesized according to a modification of the method previously reported and patented (Gazzaniga et al., 2006). CHA nanocrystals have been allowed, after synthesis, to grow in the reaction mixture under stirring up to the formation of nanostructured clusters having dimensions ranging from about 0.5 to 3.0 μm, according to the patented methodology. Then, the stirring was suspended allowing the deposition of CHA nanostructured microclusters isolated by filtration of the solution, repeatedly washed with water, and freeze-dried (Roveri et al., 2009b). CHA nanocrystals as well as the formed microclusters were analyzed by means of SEM, TEM, XRD, FT-IR. Specimens of human enamel served as controls.

### Morphological characterization

Scanning Electron Microscopy (SEM) observations were carried out by means of a SEM, Carl-Zeiss EVO, 40 XVP (Oberkoche, Germany) equipped with energy dispersive detector (EDAX) Inca 250 (Oxford, UK), using secondary electrons at 25 Kv and different magnifications. Specimens were mounted on aluminum stubs with a carbon tape and covered by a 10 nm thick carbon coating, using a coating unit.

Transmission Electron Microscopy (TEM) investigations were carried out by means of a Philips CM 100 (Eindhoven, The Netherland) instrument. The powdered samples were ultrasonically dispersed in ultra pure water and then a few droplets of the slurry deposited on holey-carbon foils were mounted on conventional copper micro grids. This latter morphological characterization was realized only on CHA nanocrystals and CHA nanostructured microclusters contained in the Zn-CHA experimental toothpaste.

### X ray diffraction analysis (XRD)

X-ray diffraction powder patterns were collected using an Analytical X'Pert Pro (Eindhoven, The Netherland) equipped with X'Celerator detector powder diffractometer using Cu Kα radiation generated at 40 kV and 40 mA. The instrument was configured with a 1° divergence and 0.2 mm receiving slits (X'PERT Guide, 2004).

The samples were prepared using the front-loading of standard aluminum sample holders, which are 1 mm deep, 20 mm high and 15 mm wide. The crystallinity degree was evaluated according to the following formula: crystallinity = (X/Y) 100 where X = net area of diffracted peaks, and Y = net area of diffracted peaks + background area.

### Infrared microscopy spectral analysis (FT-IR)

FT-IR spectra were recorded on a Thermo Nicolet 380 FT-IR spectrometer (Illinois, USA) equipped with a commercial ATR accessory. The infrared spectra were registered from 4000 to 400 cm^−1^ at 2 cm^−1^ resolution using a Bruker IFS 66v/S spectrometer using KBr pellets. Spectra were collected by averaging 32 scans at 4 cm^−1^ resolution.

## Results

### Carbonate-hydroxyapatite (CHA) nanocrystals and CHA microclusters characterization

**Figure 1:**
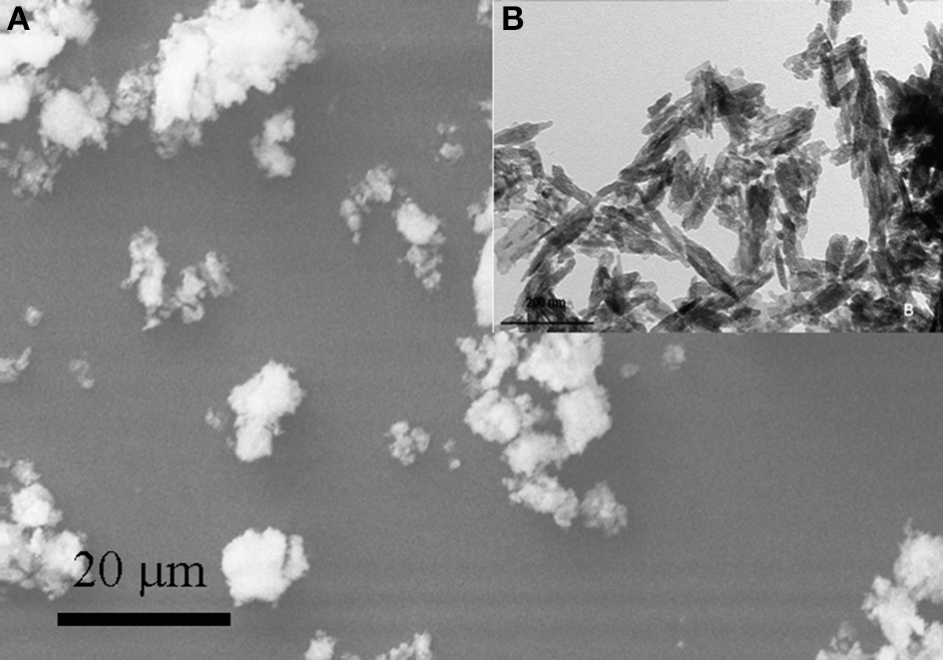
**Scanning electron micrograph of synthetic biomimetic CHA nanocrystals forming microclasters (A), and the inset showing the transmission electron micrograph of CHA nanocrystals with characteristic acicular morphology (B)**.

Figure 1 shows scanning and transmission electron micrographs of synthetic biomimetic CHA nanocrystals, with characteristic plate-like features and acicular morphology, and the aggregated CHA nanostructured microclusters.

**Figure 2:**
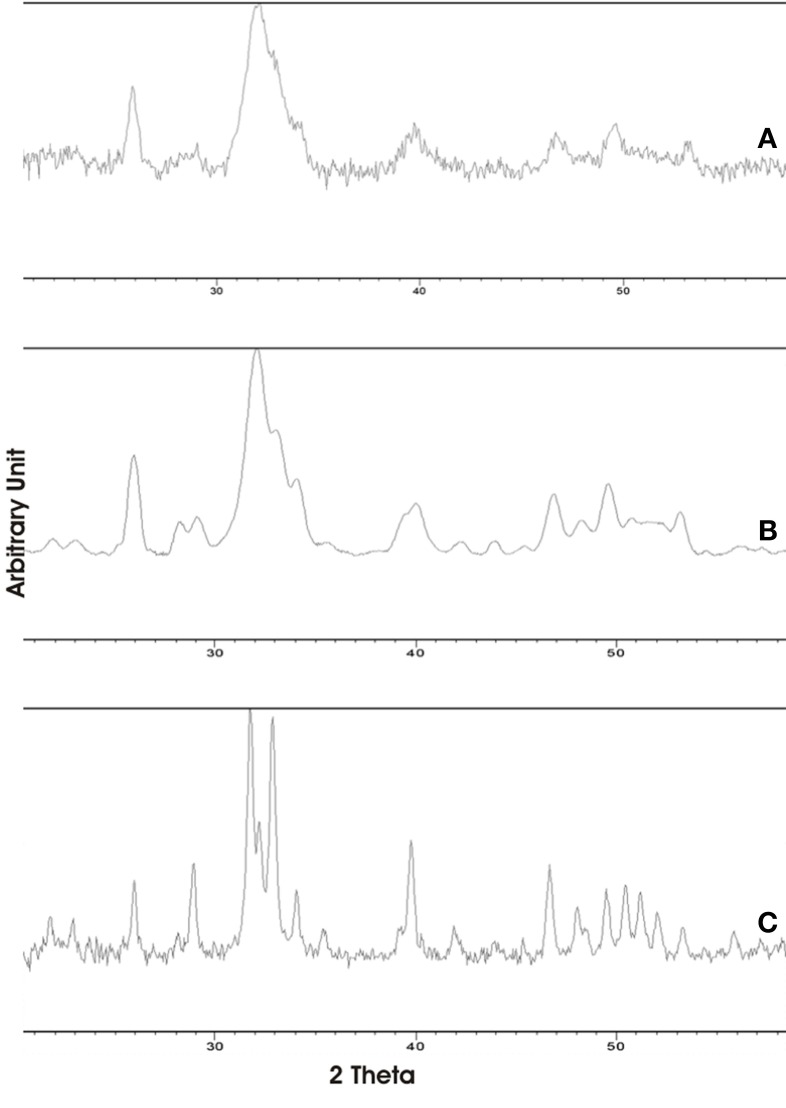
**X-ray diffraction patterns collected for synthetic biomimetic CHA nanocrystals (A), CHA nanostructured microcrystals (B), and human tooth enamel (C)**.

**Figure 3:**
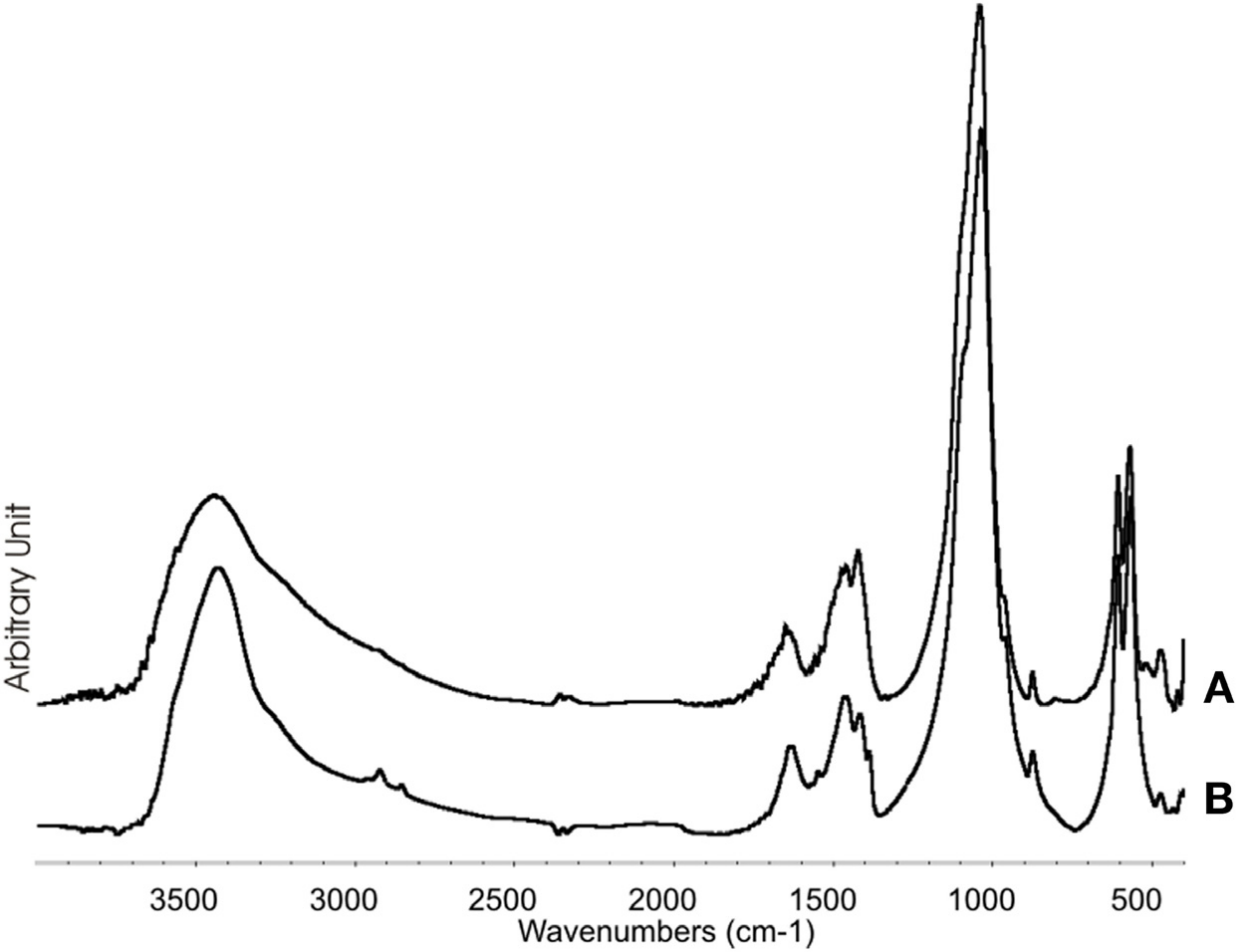
**FT-IR Synthetic biomimetic CHA nanostructured microclusters (A), and natural enamel (B)**.

The XRD of synthetic biomimetic CHA nanocrystals, showing the broadened diffraction maxima of an apatite single phase, is reported in Figure 2A. This pattern is compared with the XRD collected from the synthetic biomimetic CHA nanostructured microclusters used for preparing the Zn-CHA toothpaste (Figure 2B) and the XRD of human tooth enamel (Figure 2C). There are evident similarities between Figures 2A,B, whereas Figure 2C shows the highest degree of crystallinity, proper of the natural enamel. The FT-IR spectra of synthesized CHA nanostructured microclusters and human enamel apatite are reported in Figures 3A,B, respectively. In both these spectra, it is possible to find characteristic bands at 1032 - 1104 cm^−1^, due to the phosphate group. The absorption band at 1472 cm^−1^ is related to the carbonate group substitution of the phosphate one, while the shoulder at 1550 cm^−1^ can be attributed to the carbonate group substituting the hydroxyl group in the apatite structure.

### Enamel surface analysis

A total of 12 teeth (one for each subject) were evaluated: five teeth extracted from subjects of the experimental group after the protocolled use of the Zn-CHA toothpaste, five teeth from subjects of the active control group after the protocolled use of KNO_3_/NaF toothpaste, two teeth from subjects that did not participate to the protocol (that brushed their teeth with a non-specified fluoride toothpaste, negative control). No complications after the extractions were observed in all the subjects enrolled in the present morphological *in vivo* investigation.

**Figure 4:**
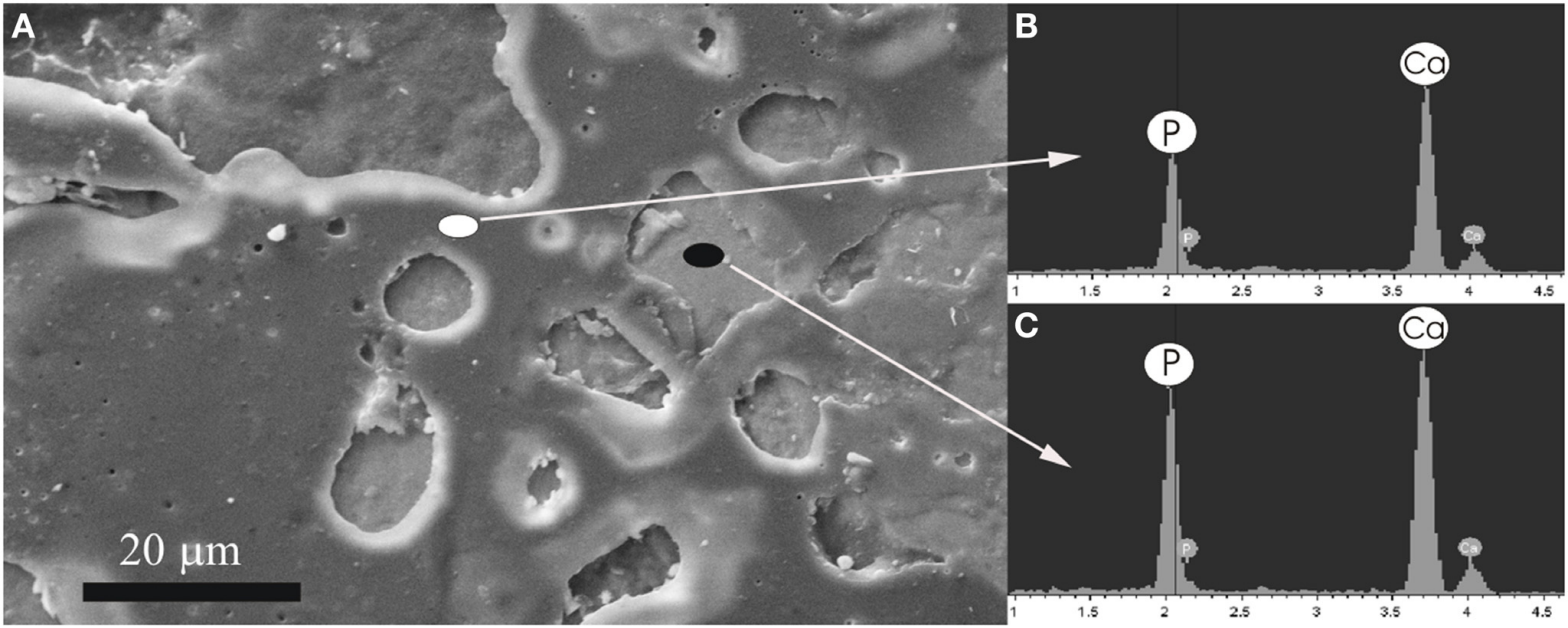
**Scanning electron micrograph of the enamel surface of a tooth *in vivo* treated using the Zn-CHA toothpaste (A), EDAX results using a probe pointed in the area labeled by the white circle (B), EDAX results using a probe pointed in the area labeled by the black circle (C)**.

Figure 4A shows a scanning electron micrograph of the enamel surface of a tooth treated with Zn-CHA toothpaste. In this micrograph, two different enamel superficial morphologies are evident: homogeneous smooth surface areas (black circle), characterized by scraped surfaces, and areas of thin depositions (white circle). Figures 4B,C report the elementary analysis results obtained by EDAX probe pointed on surface areas labeled by black and white circles, respectively. No evidence of silica and fluoride can be observed, only the presence of calcium (C) and phosphorus (P). The Ca/P molar ratio results, calculated from Figures 4B,C, are 1.67 and 1.63, respectively.

**Figure 5:**
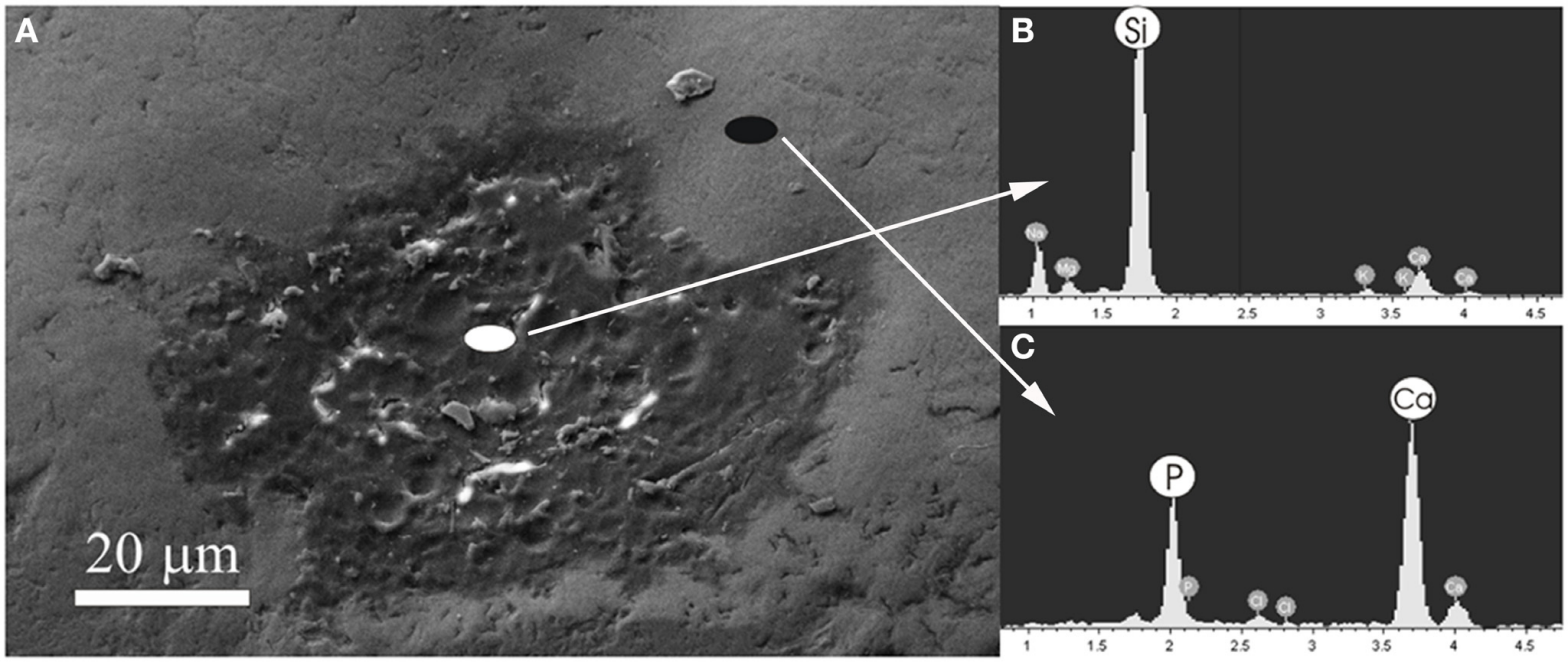
**Scanning electron micrograph of the enamel surface of a tooth *in vivo* treated using the KNO_3_/NaF toothpaste (A), EDAX with probe pointed in the area labeled by the white circle (B), EDAX with probe pointed in the area labeled by the black circle (C)**.

Figure 5A shows the scanning electron micrograph of the enamel surface of a tooth treated using KNO_3_/NaF toothpaste (positive control), in which two different areas can be discerned. In the first central area (white circle), there is a deposition of a phase mainly composed by silica and sodium, as shown in the elementary analysis results obtained by EDAX probe (Figure 5B). The presence of fluoride has not been detected. The elementary analysis results obtained by EDAX probe of the other area (black circle) (Figure 5C) reveal a Ca/P molar ratio of 1.9, which is the characteristic value of the native enamel hydroxyapatite (Wirsing et al., 1974; Hoyer et al., 1984).

**Figure 6:**
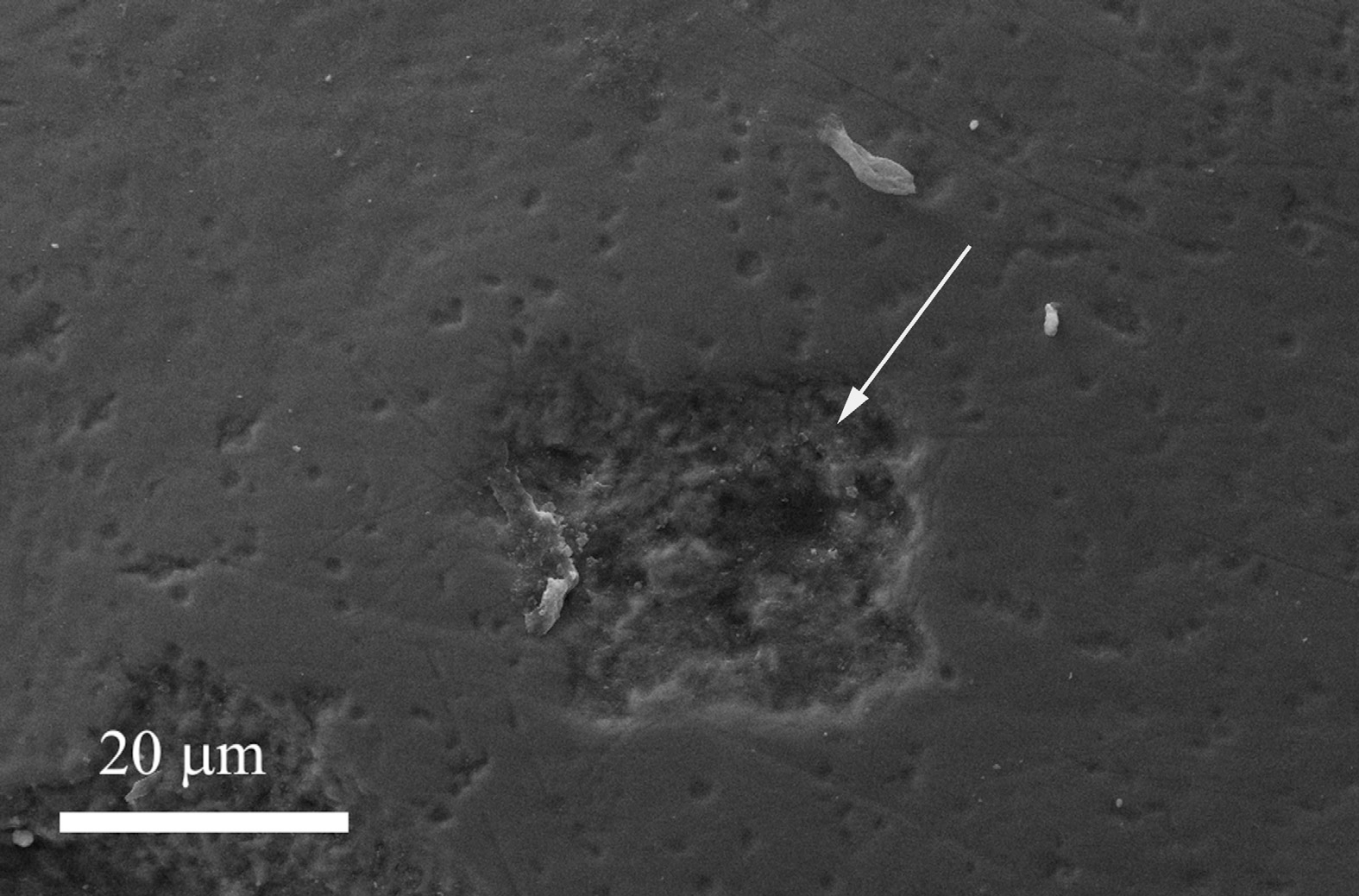
**Scanning electron micrograph of the enamel surface treated using a non-specified fluoride toothpaste, showing a zone of enamel loss (arrow)**.

The enamel surface of a tooth representing the negative control, brushed with a non-specified fluoride toothpaste shows a zone of enamel loss (Figure 6).

**Figure 7:**
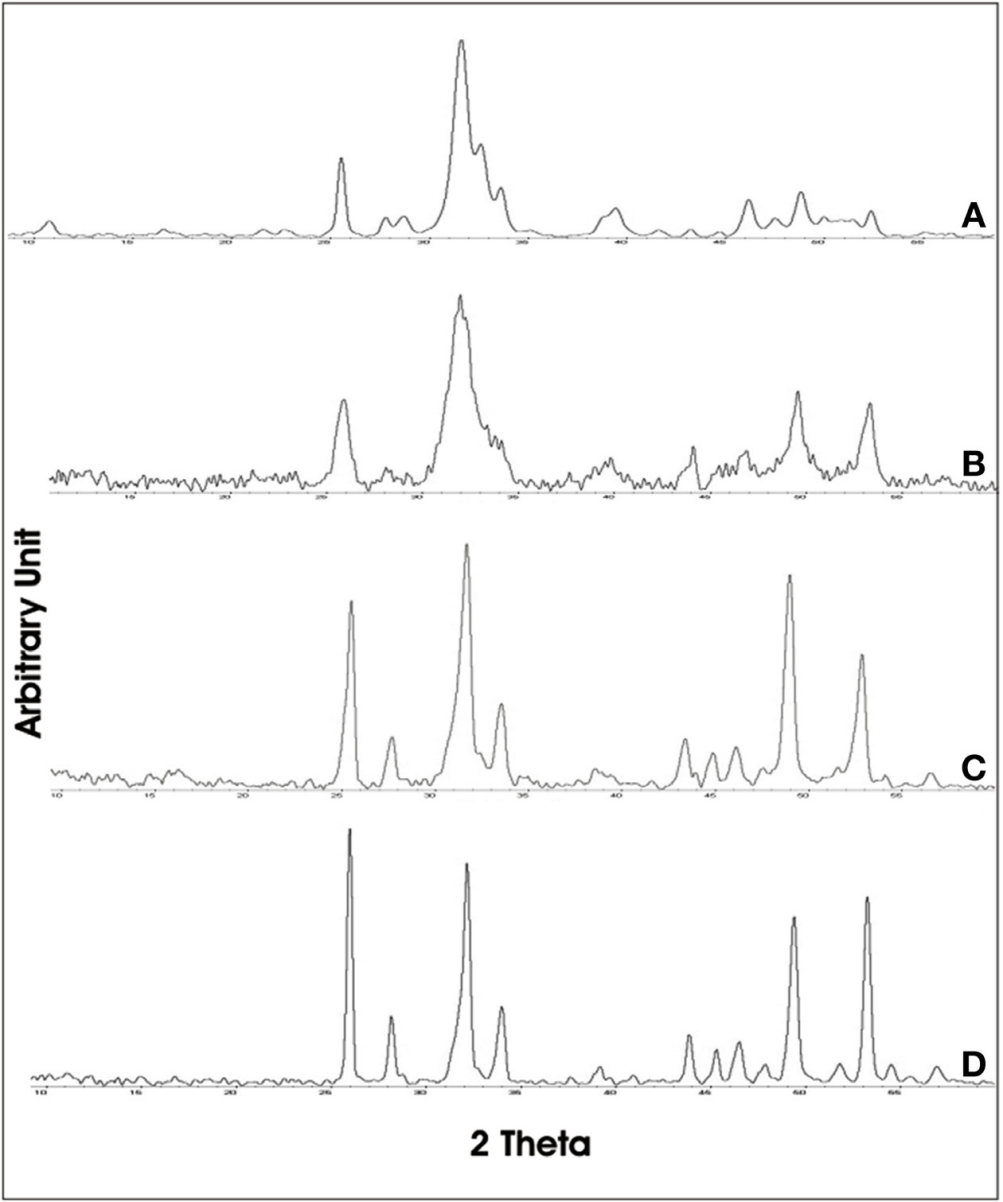
**DRX pattern of the enamel after the *in vivo* treatment using the following. (A)** synthetic biomimetic CHA nanostructured microclusters, **(B)** Zn-CHA toothpaste, **(C)** KNO3/NaF toothpaste, **(D)** Fluoride toothpaste.

Figure 7A shows the broadened X-Ray diffraction maxima of the synthetic biomimetic CHA microclusters of the Zn-CHA experimental toothpaste. This XRD pattern can be compared with the ones obtained from enamel slabs of teeth treated using Zn-CHA toothpaste, KNO_3_/NaF toothpaste and fluoride toothpaste, reported in Figures 7B–D, respectively. The XRD pattern obtained from enamel surfaces treated using Zn-CHA toothpaste (Figure 7B) is similar to the one obtained from Zn-CHA nanostructured microclusters (Figure 7A), thus revealing its deposition on the enamel surface.

**Figure 8:**
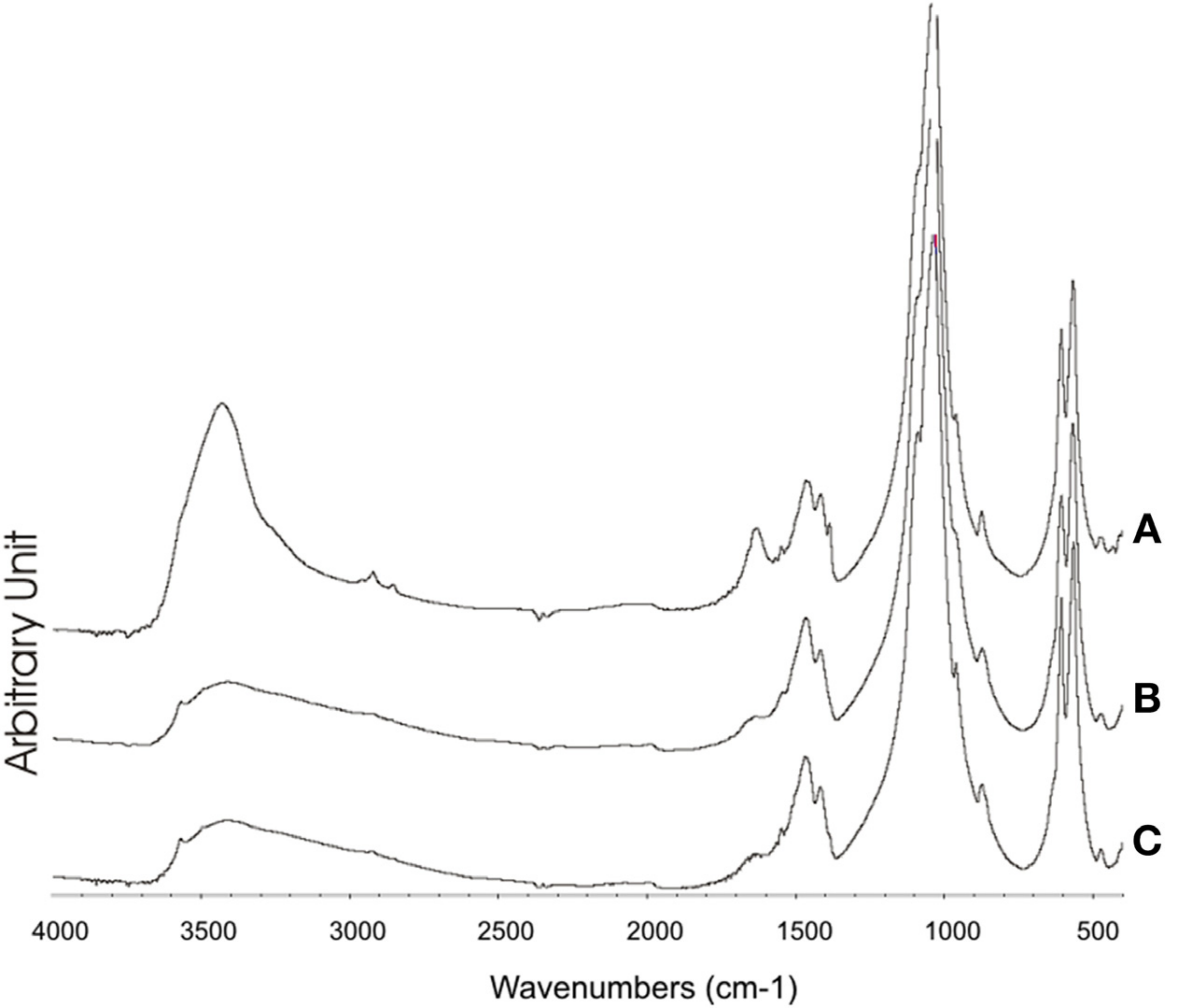
**FT-IR of the enamel surface after treatment using. (A)** Zn-CHA containing toothpaste, **(B)** KNO_3_/NaF toothpaste, **(C)** Fluoride toothpaste.

On the other hand, the XRD pattern recorded on the enamel surfaces obtained from teeth treated using KNO_3_/NaF toothpaste (Figure 7C) appear slightly less sharpened than those obtained from the surface of enamel treated using the fluoride toothpaste (Figure 7D). Figure 8A shows the FT-IR absorption pattern of the enamel surface of teeth treated with Zn-CHA toothpaste, which is similar to the one of the enamel HA (Roveri et al., 2009b).

The analysis of the FT-IR absorption phosphate bands at about 1030 - 1090 cm^−1^ shows the different degree of crystallinity relative to the different enamel samples analyzed. In fact, the adsorption bands reported in Figure 8A demonstrate a low degree of resolution of the phosphate peak in the enamel treated using the CHA-based toothpaste. On the other hand, the FT-IR absorption pattern obtained in the enamel treated using the KNO_3_/NaF toothpaste (Figure 8B) reveals the presence of a peak at 3573 cm^−1^, which is characteristic of the substitution of hydroxyl groups with fluoride ions, into the hydroxyapatite structure (Wang et al., 2009). This peak is also visible in the FT-IR absorption pattern obtained in the spectrum of enamel *in vivo* treated with the fluoride toothpaste (Figure 8C).

## Discussion

Hydroxyapatite, in enamel and in bone, is responsible for the mechanical behavior of the calcified tissues. Unlike bone, when the enamel hydroxyapatite is dissolved or abraded, it cannot spontaneously remineralize, because enamel contains no cells (Roveri et al., 2009a). Biomimetic carbonate hydroxyapatite (CHA) nanocrystals have been synthesized with a stoichiometric Ca/P molar ratio of about 1.7 ± 0.1, containing 4 ± 1 wt% of carbonate ions, prevalently replacing phosphate groups, while 1% of Ca^2+^ ions are substituted by Zn^2+^. The nanostructured Zn-CHA microcrystals, prepared in laboratory according to the patented methodology, represent the active component of the experimental Zn-CHA toothpaste. The micrometric dimension of the crystal clusters allows avoiding any suspicion about the *in vivo* utilization of nano-dimensioned particles. Nevertheless, the nanostructured surface of the microclusters is responsible for the high surface area that is crucial for their chemical reactivity.

Synthesized biomimetic CHA nanocrystals and human enamel apatite not only contain a similar carbonate amount, but also have been shown to promote carbonate substitution to the phosphate and/or hydroxyl group, which is very similar to the synthetic and biological CHA nanocrystals. The synthetic experimental CHA nanocrystals have a plate-like morphology and a structure very close to that of the enamel, dentine and bone hydroxyapatite, and exhibiting very similar FT-IR spectra, even if enamel spectrum shows the highest degree of crystallinity according to previous findings (Teaford, 1988).

In summary, in the present work, the surface of teeth treated *in vivo* for 8 weeks with: (i) Zn-CHA toothpaste (experimental group); (ii) KNO_3_/NaF toothpaste (active control group); (iii) fluoride toothpaste (negative control group) have been compared, by means of SEM-EDAX investigation, XRD, and FT-IR.

SEM-EDAX, XRD, and FT-IR observations showed that, after the *in vivo* treatment using the Zn- CHA toothpaste, an appreciable formation of a biomimetic CHA coating was present on the enamel surface. On the other hand, not any mineral deposition, in spite of silica, has been observed after the *in vivo* treatment using both a KNO_3_/NaF toothpaste and non-specified fluoride toothpaste. The *in vivo* treatment with these two latter toothpastes changes the structure of enamel natural hydroxyapatite crystals, increasing their degree of crystallinity (XRD investigation), by means of the partial substitution of hydroxyl groups with fluoride ions (FT-IR investigation). Indeed, the phosphate band in the enamel surface after treatment with KNO_3_/NaF or a non-specified fluoride toothpaste appears highly solved respect to the one of the phosphate group reported in Figure 8A, thus demonstrating the high degree of crystallinity of these enamel surfaces.

On the other hand, the remineralizing/repairing effect of the enamel surface treated using synthetic nanostructured CHA microcrystals is consistent with a mineral biomimetic apatitic deposition, which does not alter the chemical-physic properties of the enamel. The biomimetic CHA coating can appear of different thickness, probably due to the underside different enamel surface morphology, which can change in function of the degree of enamel damage. However, the EDAX analysis reveals that the Ca/P molar ratio of CHA crystals (about 1.7) is homogeneously constant on the enamel surface. This finding assures a uniform enamel protection against the enamel wear and loss phenomena, thus preventing dentine exposure.

Results of the first clinical randomized trial by Orsini et al. (2010) have already demonstrated the efficacy of Zn-CHA toothpastes in reducing DH. Moreover, a further very recent randomized trial by the same authors showed that this effect could be exerted after only 3 days of treatment (Orsini et al., 2013).

The results of this *in vivo* morphological and chemical-physic study might in part explain the beneficial effect of Zn-CHA toothpastes in reducing DH, since the deposition of a synthetic nanostructured CHA microcrystals-rich coating could lead to a remineralizing/repairing effect of the enamel surface, in the teeth treated using Zn-CHA toothpaste. Therefore, the principal finding of this study is that: (1) the remineralizing mechanism of the nanostructured CHA microcrystals, largely documented by previous *in vitro* reports (Roveri et al., 2008, 2009a) can be also confirmed *in vivo*. Moreover, it can be suggested that this synthetic CHA deposition mainly occurs on the enamel areas characterized by enamel loss and/or damage (probably due to erosion effects), thus being considered as a real enamel repair (see Figure 4).

In contrast, (2) the use of a toothpaste containing KNO_3_/NaF may form only a deposition, consisting of silica, as an abrasive phase on the enamel surface, which, however, does not remineralize the damaged enamel area, but it is generally deposed in correspondence of natural concavities proper of the natural tooth morphology (see Figure 5). Furthermore, no deposition on the enamel surfaces has been observed after treatment using the fluoride and the KNO_3_/NaF toothpastes (see Figure 6), which may lead only to a partial substitution of the hydroxyl groups with fluoride ions in the native enamel hydroxyapatite.

The CHA formed coating is generally insoluble in physiological mouth pH, but it may undergo to solubilization when, for instance, a bacterial biofilm covers the teeth and its products decrease the pH value. During the CHA coating solubilization, Ca ions, phosphates, and Zn ions are released, allowing the Zn to exploit a strong antibacterial effect, which interferes with the plaque formation, thus preventing further solubilization processes of the newly deposed Zn-CHA coating. Therefore, it may be suggested that the coating formed by Zn- CHA toothpastes may exploit not only a remineralizing effect of the dental surface, but also a beneficial effect toward bacterial plaque attacks.

The main limitation of this work is that the *in vivo* remineralizing effect exploited by the Zn-CHA toothpaste was morphologically demonstrated only on the enamel surfaces, since the analyzed extracted teeth did not present areas of dentine exposition. Therefore, further studies will be carried out *in vivo* to analyse whether a stable biomimetic CHA deposition (Roveri et al., 2009a) and a repairing mechanism can be demonstrated also in dentinal surfaces.

In conclusion, the present study shows that only the use of a toothpaste containing Zn-substituted CHA nanocrystals can produce a biomimetic coating on the enamel surface, thus mimicking the composition, structure, morphology and surface reactivity of the biological enamel hydroxyapatite.

### Conflict of interest statement

The authors declare that the research was conducted in the absence of any commercial or financial relationships that could be construed as a potential conflict of interest.
